# A pilot study of occupational exposure to ultrafine particles during 3D printing in research laboratories

**DOI:** 10.3389/fpubh.2023.1144475

**Published:** 2023-06-02

**Authors:** Giorgio Felici, Joanna Izabela Lachowicz, Simone Milia, Emanuele Cannizzaro, Luigi Cirrincione, Terenzio Congiu, Mariusz Jaremko, Marcello Campagna, Luigi Isaia Lecca

**Affiliations:** ^1^Department of Medical Sciences and Public Health, Division of Occupational Medicine, University of Cagliari, Cittadella Universitaria, Cagliari, Italy; ^2^Department of Sciences for Health Promotion and Mother and Child Care “Giuseppe D’Alessandro”, University of Palermo, Palermo, Italy; ^3^Smart-Health Initiative (SHI) and Red Sea Research Center (RSRC), Division of Biological and Environmental Sciences and Engineering (BESE), King Abdullah University of Science and Technology (KAUST), Thuwal, Saudi Arabia

**Keywords:** 3D printing, nanoparticles, indoor pollutants, fused filament fabrication, stereolithography

## Abstract

**Introduction:**

3D printing is increasingly present in research environments, and could pose health risks to users due to air pollution and particulate emissions. We evaluated the nanoparticulate emissions of two different 3D printers, utilizing either fused filament fabrication with polylactic acid, or stereolithography (SLA) with light curing resin.

**Methods:**

Nanoparticulate emissions were evaluated in two different research environments, both by environmental measurements in the laboratory and by personal sampling.

**Results:**

The SLA printer had higher nanoparticulate emissions, with an average concentration of 4,091 parts/cm^3^, versus 2,203 particles/cm^3^ for the fused filament fabrication printer. The collected particulate matter had variable morphology and elemental composition with a preponderance of carbon, sulfur and oxygen, the main byproducts.

**Discussion:**

Our study implies that when considering the health risks of particulate emissions from 3D printing in research laboratories, attention should be given to the materials used and the type of 3D printer.

## Introduction

3D printing, also known as additive manufacturing (AM), is a cutting-edge technology that offers countless design possibilities and has seen a growing number of applications in industrial, educational, and home settings. 3D printing is widely used and is known to release inhalable ultrafine particles (UFP) and volatile organic compounds (VOCs) (1–5). The scientific community has therefore begun to have concerns about its possible health implications and aims to identify related toxicological risks from both occupational and domestic exposure. Domestic settings in particular may not have adequate ventilation systems to deal with pollutants.

There are numerous gaps in our understanding of UFP emissions and their biochemical behavior in the atmosphere and the human body. In recent years, more studies have emerged that investigate occupational exposure (6), with a focus on the biological monitoring of individual workers (7).

UFPs are particles with an aerodynamic diameter of less than or equal to 0.1 microns. They constitute the smallest dimensionally fraction of particulate matter (PM) and are also the most abundant component.

In contrast to particulate PM_10_ and PM_2.5_, UFP is characterized by a greater ability to reach the most distal regions of the respiratory system (alveoli), greater ability to evade the lung’s primary defense systems, and the ability therefore to creep through the alveolus-capillary barrier, eventually reaching the circulatory system and the whole body (8, 9).

This ability of UFPs to penetrate deep into the lung and enter the bloodstream may account for the cardiovascular effects caused by exposure to these particles. These effects include altered coagulation, damage of the vascular endothelium, and altered heart rate (10), with increased markers of thrombosis and inflammation in the blood circulation, and a reduced heart rate variability index (11–13). Previous works has shown that the emission of particulate matter and air pollutants of different physical (size, shape, concentrations) and chemical types is influenced not only by the printer type, but also by the materials used for additive manufacturing (e.g., the chemical composition and melting temperatures) (14). Some studies have revealed that even using one printing procedure, there can be variations in emissions when using different colors of the same material (15).

Especially in the scientific and industrial fields, printing materials can be altered with other components such as metals, to improve their chemical and physical characteristics (16). This may also change the exposures and risks related to their use, however.

Growing awareness of the chemical and physical properties of nanoparticles, coupled with the use of monitoring that focuses both on the environment and, as much as possible, on individuals, could be beneficial for future research. This could help to identify early biological effects, and possible synergistic and additive mechanisms when other risk factors are present. It may also allow the development of new protective measures against these exposures.

The objective of this pilot study is to measure the occupational exposures to UFP in research environments, and therefore assess the risks associated with the use of different kinds of 3D printers.

## Materials and Methods

### Study design and sampling environment

The study was conducted in two phases. The first phase was on the 22nd of September, in the Einstein Telescope Laboratory of the National Institute of Nuclear Physics (INFN) [39.27085612372698, 9.122284443006196]. Environmental monitoring of UFPs was carried out using an Ultimaker S5 (technical data available in Electronic Supplementary material), a desktop 3D printer produced by the Ultimaker company that uses fused filament fabrication (FFF) technology for printing. The material used for printing was a silver filament of polylactic acid (PLA), 2.85 mm in diameter, produced by Ultimaker. PLA is a biopolymer obtained from agricultural crops that is used in various fields such as the biomedical, textile, or packaging industries (17).

Sampling was carried out during simultaneous printing of four components with 10% corrugated infill lasted 3 h, build platform temperature was maintained in the range of 30°C to 60°C, while printer nozzle worked at 215°C.

The laboratory was a room with height, width and depth of 3.24 m, 8.44 m and 6.03 m, respectively. The room had a ducted cold-air ventilation system in operation throughout the study, to minimize possible external interference. It also had three windows that remained closed for the duration of the monitoring.

The second monitoring phase was held on September 29th, at the Department of Mechanical, Chemical and Materials Engineering at the University of Cagliari [39.273216786105486, 9.126163117412798]. This department featured a Form 2, a desktop stereolithographic (SLA) 3D printer produced by Formlabs. This printer is normally used here for 1:1 printing of orthopedic CT reconstructions, which are useful to understand patient conditions and tailored solutions.

This printing technology uses liquid resins that cure when exposed to light of certain wavelengths. It involves two additional post-printing steps. The first step consists of washing the molded part in a tank with isopropyl alcohol or tripropylene glycol monomethyl ether to remove any resin residue. The second step is carried out in a curing chamber, that allows the product to finish curing through a combined exposure to light and heat (UV emissions).

In this case, continuous environmental monitoring of these steps was performed starting with a 2 h25’ print of 8 bases for device applications on skin, using a resin produced by Form 2. The solvent used for washing was isopropyl alcohol. Temperature reached for curing phase was of 60°C with a LED wavelength was of 405 nm. This laboratory was a room with a height, width and depth of 3.12 m, 7.6 and 6.85 m, respectively. The windows were closed and there was no ventilation system operating.

### Procedures

**Figure 1:**
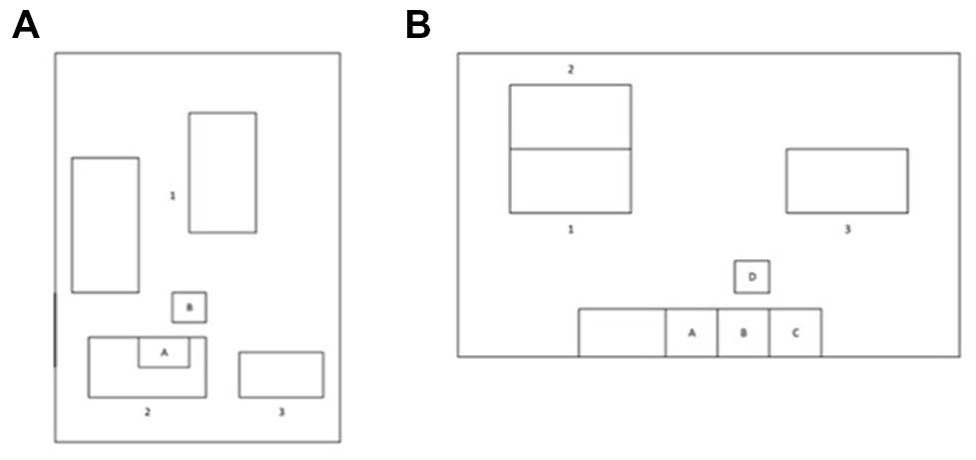
Layout of the research environments sampled in this study. **(A)** Layout of the room at the Einstein Telescope Laboratory. A: 3D Printer, B: Low Pressure Electric Impactor (ELPI+). The numbers 1, 2 and 3 represent the positions of workstations occupied by participants. **(B)** Layout of the room at the Department of Mechanical, Chemical and Materials Engineering, University of Cagliari. A: 3D Printer, B: washing tank, C: curing chamber, D: ELPI+. The numbers 1, 2 and 3 represent the positions of workstations occupied by participants.

In both scenarios, UFP exposure assessment was performed with personal and environmental measurements in real-life office conditions and while activities occurred inside the laboratories during the 3D printing process. During the test days two different type of sampling were performed simultaneously. The first was environmental sampling with continuous monitoring, lasting at least 4 h. The second was personal exposure assessment, lasting about 1 h per participant. Tested subjects never left the rooms where printers were operating during the entire duration of sampling. UFPs were collected in real time through two instruments, namely DiSCmini (measures the personal exposure and is placed physically closed to the nose and mouth of the participant) and ELPI+™ (measures the environmental exposure) as described in Figure 1. The participants were not using protection devices.

### Personal UFP Monitoring

Three participants underwent personal monitoring of UFPs in the two different days and settings (September 22nd for the first setting and September 29th for the second setting). All subjects were male, with a mean age of 33 years (range: 27 to 41 years).

In the first setting, environmental monitoring lasted for 4 h 2 min, while the three participants underwent personal monitoring for 2 h 43 min 55 s (respectively 50, 51 and 62 min in succession). In the second setting, environmental monitoring lasted 4 h 2 min, with individual monitoring lasting 3 h 59 s (60, 59 and 61 min for each participant in succession). There was no data loss.

Personal sampling of particulate matter was performed by the Diffusion Size Classifier (DiSCmini - Testo SE & Co. KGaA, Milan, Italy; Technical data available in Electronic Supplementary material). This is based on unipolar diffusion charging of particles followed by detection in two electrometer stages to determine NPs number, average diameter and lung-deposited surface area (LDSA) concentration. The DiSCmini particles detection range is 10 to about 300 nm with a concentration range of 10^3^ to 10^6^ particles/cm^3^. Its accuracy is +/− 30% with a flow rate of 1 L/min (18, 19). The following UFP parameters were collected: UFP concentration as number of particles/cm^3^; mean size (nm); LDSA concentration (μm^2^/cm^3^); total LDSA expressed as a cumulative dose (μm^2^). LDSA dose (modeled values) was calculated with the following formula:

Total LDSA = mean LDSA × V.

where LDSA is the lung deposition surface area in μm^2^/cm^3^, and V is the sampled volume in liters.

DiSCmini was calibrated by the manufacturer. The proper DiSCmini functioning was verified by the contemporary analysis with ELPI+ at the same experimental conditions.

### Environmental UFP Sampling

UFP environmental sampling used a low-pressure electric impactor model ELPI+™ (Electric Low Pressure Impactor—Dekati Ltd., Kangasala, Finland^1^;). This device measures in real time the concentration and size distribution of particles. It has a range diameter of 0.006–10 μm and was operating at a nominal flow rate of 10 L/min. The greatest advantage of ELPI+™ is sample collection by 14 channels, which select PMs of different size. The filer box was opened under the sterile laminar flow head and placed for each channel in the impactor. The selected particle size is as follows: channel 1 (0.006 μm), channel 2 (0.017 μm), channel 3 (0.031 μm), channel 4 (0.055 μm), channel 5 (0.095 μm); channel 6 (0.156 μm); channel 7 (0.258 μm); channel 8 (0.384 μm), channel 9 (0.606 μm), channel 10 (0.952 μm), channel 11 (1.640 μm), channel 12 (2.480 μm), channel 13 (3.670 μm) and channel 14 (5.390 μm).

The ELPI+™ was connected to an air intake pump with a flow rate of 0.6 m^3^/h and a pressure of 40 mbar at the final stage of the impactor (absolute filter). Though UFP have diameter < 100 nm, for the purpose of this paper, this concentration was calculated as the sum of particles with a Central geometric mean diameter (Di) between 10 nm and 314 nm. Detailed descriptions of the ELPI+™ function and its principles of operation are given in the literature (20, 21).

The complete ELPI+™ dataset for each laboratory is available as Supplementary material.

### Chemical analysis: SEM and X-ray micronalysis

The particulate matter was collected with an ELPI on Whatman Nucleopore Track-Etch membranes (part number 800203, PC MB 25 mm, no holes). The following channels were used for PM collection: 5 (0.095 μm); 6 (0.156 μm); 7 (0.258 μm); 8 (0.384 μm) and 11 (1.640 μm). The time of PM collection corresponds to the ELPI sampling time.

Small pieces of each filter were affixed to aluminum SEM stubs. Every sample was coated with gold using a Cressington 108auto Sputter Coater to achieve conductivity. The morphology and elemental composition of the particulate matter was analyzed with a scanning electron microscope (SEM) (Sigma 300, Zeiss, Oberkochen, Germany) in combination with energy dispersive X-ray spectrometry (EDX XFlash detector 630 M, Bruker Nano Gmbh, Berlin Germany) (22).

## Results

Table 1 shows the results of personal UFP exposure monitoring of the study participants in the two study settings. UFP concentration means were 2,203 and 4,091 part/cm^3^ (SD = 538 and 429, respectively)., while total LDSA means were 424 mm^2^ for the first setting and 667 mm^2^ for the second setting (SD = 90 and 79, respectively).

**Table 1:** UFP parameters from personal sampling in the two study settings.

Setting 1 UFP Parameters (*N* samples = 3)			
UFP (part/cm^3^) Mean (SD)	Size (nm) Mean (SD)	LDSA (μm^2^/cm^3^) Mean (SD)	Total LDSA (mm^2^) Mean (SD)
2,203 (538)	60 (5)	8 (1)	423 (90)
Setting 2 UFP parameters (*N* samples = 3)			
UFP (part/cm^3^) Mean (SD)	Size (nm) Mean (SD)	LDSA (μm^2^/cm^3^) Mean (SD)	Total LDSA (mm^2^) Mean (SD)
4,091 (429)	48 (3)	11 (1)	667 (79)

LDSA, lung deposition surface area; SD, standard deviation; UFP, ultrafine particles. The presented data are not background corrected.

Results of environmental exposure assessment by ELPI+ sampling during the entire 3D printing process in the two different scenarios are reported in Table 2. UFP concentration (particles/cm^3^) means were 5,414 and 11,806 with peak measurements of 8,544 and 59,152 (SD = 844 and 1966, respectively).

**Table 2:** UFP concentration (particles/cm^3^) data from ELPI+ environmental monitoring in two study settings.

Setting 1 UFP Parameters (whole environmental sampling)			
Min	Mean (SD)	Median	Max
3,290	5,414 (844)	5,360	8,544
Setting 2 UFP parameters (whole environmental sampling)			
Min	Mean (SD)	Median	Max
6,921	11,806 (1966)	12,374	59,152

The presented data are not background corrected.

**Figure 2:**
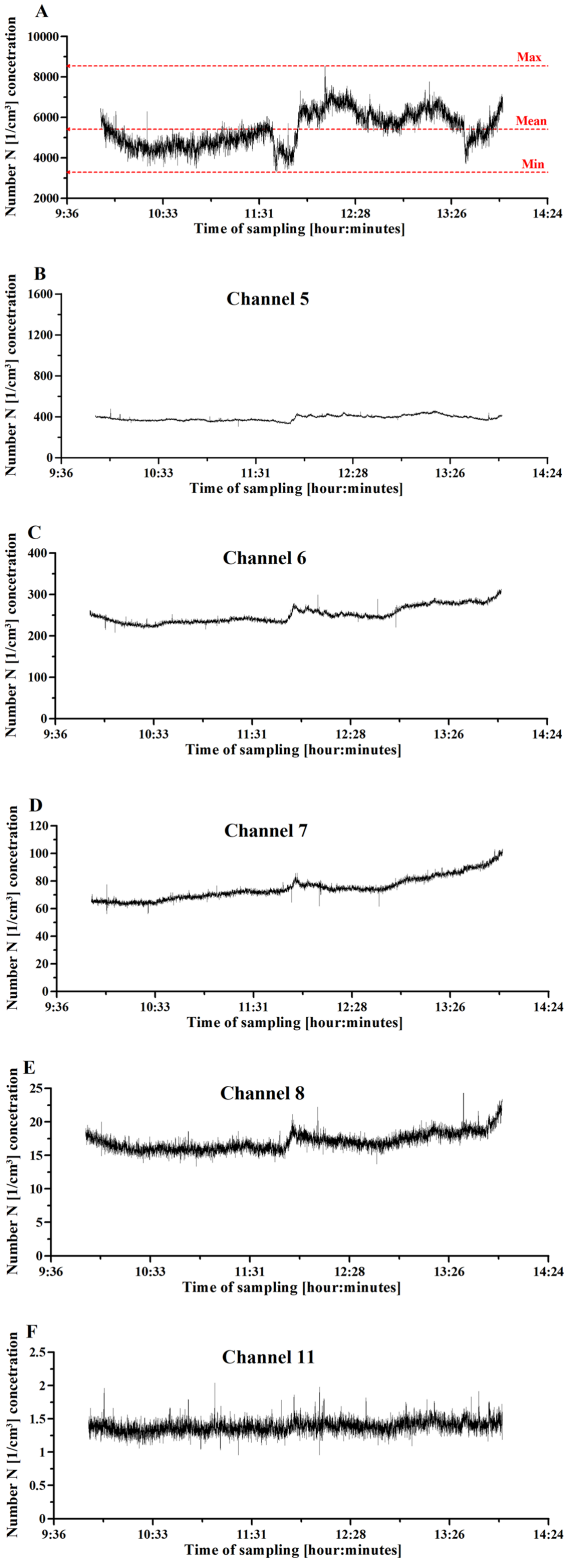
UFP concentration changes in the Einstein Telescope Laboratory setting, where 3D printing with fused filament fabrication was used. **(A)** The overall particulate matter concentration. **(B)** Concentration of particulate matter collected with channel 5. **(C)** Concentration of particulate matter collected with channel 6. **(D)** Concentration of particulate matter collected with channel 7. **(E)** Concentration of particulate matter collected with channel 8. **(F)** Concentration of particulate matter collected with channel 11. The printing activities started at 10:00 and finished at 14:00.

Figure 2 shows particulate matter concentration as a function of sampling time. The overall PM concentration profile (Figure 2A) differed from the profiles of the particulate matter collected from different channels (Figures 2B–F). Of note, the UFPs collected by the channels 1–3 (Supplementary Figure S1) contribute mainly to the total particle concentration [1/cm^3^].

**Figure 3:**
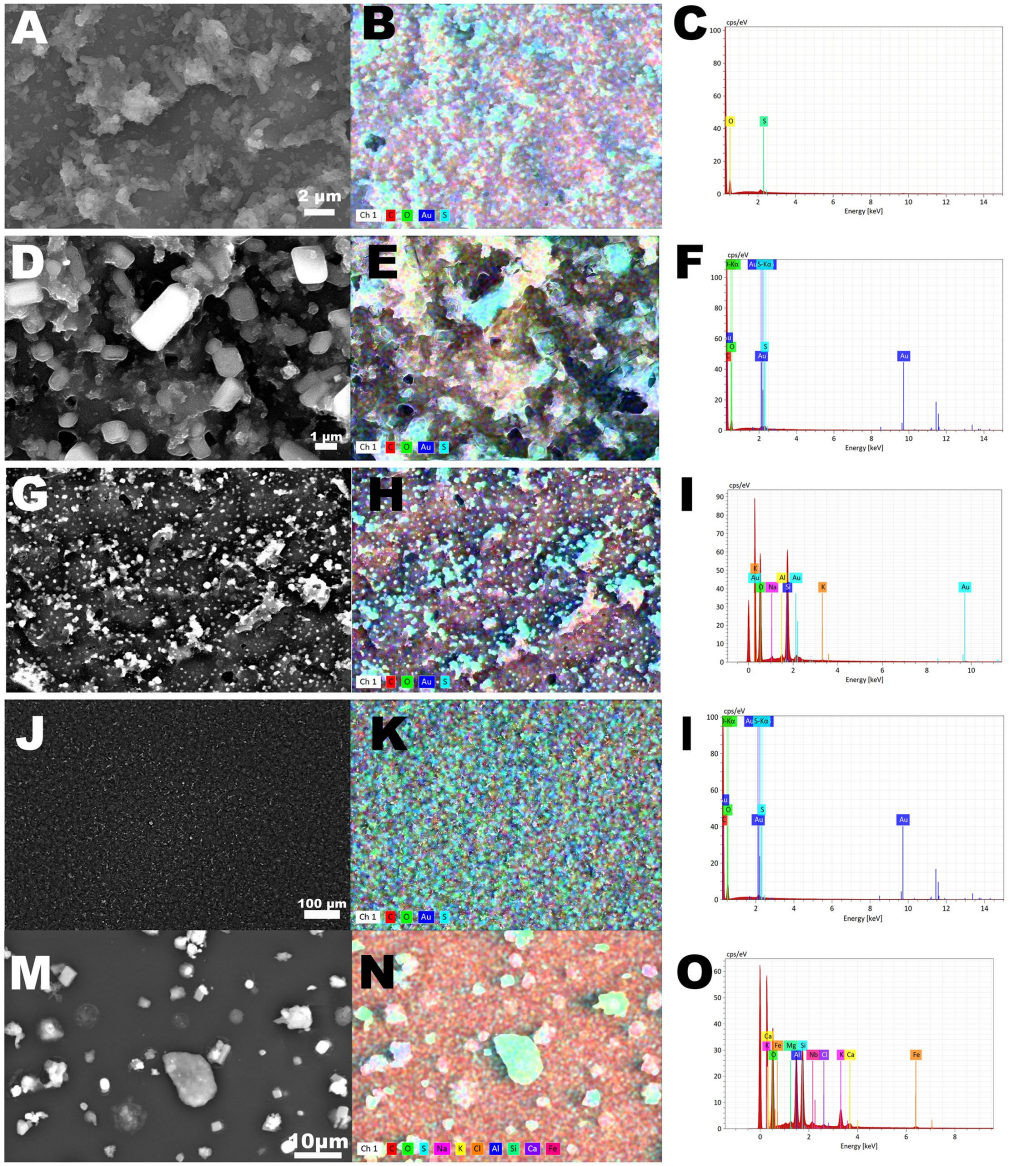
Scanning Electron Microscopy with energy dispersive X-ray spectrometry (SEM–EDX) analysis of particulate matter collected with Electric Low Pressure Impactor (ELPI+™) in Einstein Telescope Laboratory. **(A–C)** analysis of particulate matter collected on channel 5. **(D–F)** analysis of particulate matter collected on channel 6. **(G–I)** analysis of particulate matter collected on channel 7. **(J–L)** analysis of particulate matter collected on channel 8. **(M–O)** analysis of particulate matter collected on channel 11.

The morphology and elemental composition of PM collected on filters by five different channels is presented in Figure 3. While scanning, electrons are emitted from the surface, in the form of secondary electrons (SEs) and back scattered electrons (BSEs). The number of emitted electrons determines the brightness of the image on the monitor. In this analysis the emitted electrons were recorded by a specific detector, a four-quadrant semiconductor detector (QBSD). Its high sensitivity enables this detector to produce an “element contrast picture.” Heavy elements and compounds reflect more electrons than light elements, and thus appear lighter in the picture. In the EDX, signals are displayed according to the mean energy. On the X-axis of a graph, the energy is displayed in keV. The number of signals per time unit are displayed on the Y-axis, and thus the length of each line reflects the concentration of one element.

The morphology of PM collected by different channels is distinct. Particles collected in channels 5, 7 and 8 were ‘embedded’ on the filter, while particles collected by the channels 6 and 11 showed ‘crystal’ morphology. The particulate matter collected by channels 5–8 was composed mainly of carbon, sulfur and oxygen. The co-presence of suphur and oxygen on the color maps suggested the presence of sulfate in the particulate matter. The particulate matter collected by channel 11 was composed not only of carbon and sulfate, but also of sodium and chloride, potassium, aluminum and silicon, calcium and iron (Figures 3M–O).

**Figure 4:**
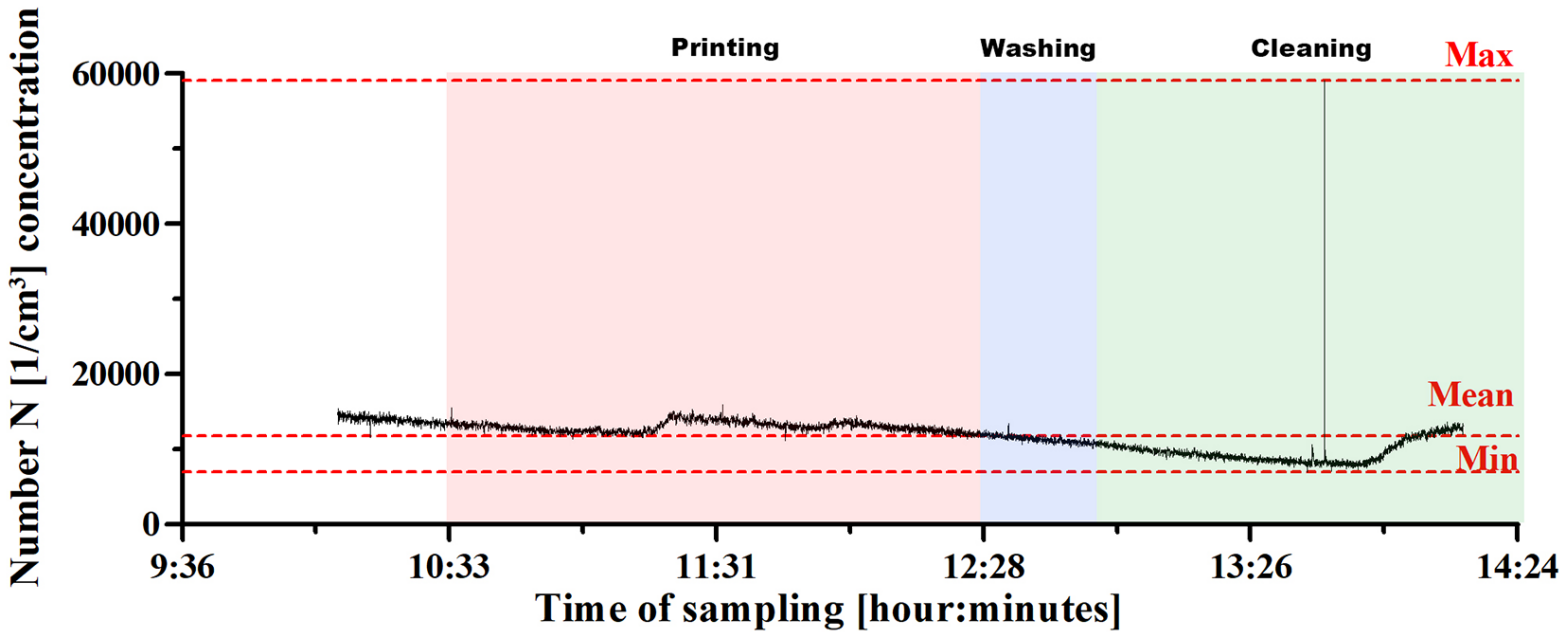
UFP concentration changes in the Department of Mechanical, Chemical and Materials Engineering at the University of Cagliari. The printing process was divided in three phases: printing, washing and cleaning.

Variations in concentration of UFP in the Department of Mechanical, Chemical and Materials Engineering at the University of Cagliari are presented in Figure 4.

## Discussion

The objective of our study was to evaluate nanoparticle exposure from different types of 3D printing. We considered different potential hazards for workers in research laboratories where desktop printers are normally used.

Unlike PM_2.5_ and PM_10,_ there are no reference limit values for UFP in the 2021 WHO “Global air quality guidelines.” (23) However, there are currently insufficient studies to date to formulate accurate air quality guidelines (AQG), making it difficult to formulate good practice advice in the literature.

This paper uses particle number concentration (PNC) as a value for quantifying environmental UFPs and indicates a low PNC for concentrations below 1,000 particles/cm^3^ (24-h mean), a high PNC for concentrations below 10,000 particles/cm^3^ (24-h mean), or 20,000 particles/cm^3^ when considering a one-hour interval. In contrast, there are no indications regarding individual exposures to UFPs.

For the FFF printer using PLA, the average environmental nanoparticulate concentration value is lower than WHO guidelines and in line with results already found in other studies for printers with the same technology (24–28).

In the case of the SLA printer, the value of the mean ambient concentration is higher than that given in the WHO guidelines with reference to the 24-h average, but still lower than the reference value for the hourly average.

Regarding these measurements, it is worth mentioning that the peak concentrations (one signal above 30,000; Figure 4) were recorded during the curing phase, when operators were preparing to remove equipment for individual exposure measurement, while the highest concentrations were normally recorded during the first printing phase.

It is also worth noting that in the second setting the background environmental measurements prior to the 3D printer being turned on were higher than those recorded in the first setting. This is similar to what was found in a study by (29) indicating the presence of inactive 3D printers and post-processing devices as a possible secondary source of UFP. Unlike their study, however, the concentrations of UFP obtained by us during printing are significantly higher.

Data of the mean concentration obtained in the second setting is difficult to compare with other studies because of the different printer models resin formulas, the specifications of which are often kept secret by manufacturers.

Table 3 shows measurements comparison with other studies of particle emission during 3D printing with FFF technology and PLA filament. The table also shows Väisänen et al. measurements for SLA printing comparison.

**Table 3:** Examples of measurements of particle emission during 3D printing.

Printers type	Particle number concentration (1× cm^−3^) or particle emission rate (1 × h^−1^)	Sampling method	References
Minifactory Oy, Finland, model 3 Education Edition Single Extruder (FFF - PLA filament)	7.4 × 10^2^ ÷ 3.7 × 10^5^ (1 × cm^−3^)	SMPS model 3,080 N (TSI), UCPC model 3,776 (TSI), SMPS series 5.400 (Grimm), PSM model A11nCNC (Airmodus Oy); CPC model 3,007 (TSI), DiSCmini (MatterAerosol AG)	Mendes et al. (24)
Cube 2nd generation, 3D Systems, Rock Hill, SC (FFF - PLA filament)	*Test chamber* 8.9 × 10^4^ (1 × cm^−3^) *Office large room* 7 × 10^2^ ÷ 2,7 × 10^3^ (1 × cm^−3^) *small room* 3,2 × 10^3^ # × cm^−3^	ASM Promo mobile (Palas), DiSCmini (MatterAerosol AG)	Steinle (28)
AFINIA-H800, Afinia, Chanhassen, MN (FFF – PLA filament)	4.2 × 10^10^ ÷ 4.2 × 10^11^ (1 × h^−1^) [*3 printers simultaneously*]	SMPS model 3,080 DMA (TSI), CPC (TSI), CPC model 3,787 (TSI), m-AMS (Aerodyne Research Inc.),model 3,787 (TSI), m-AMS (Aerodyne Research Inc.)	Katz et al. (3)
Prusa i3; Prusa Research s.r.o., Praha, Czech Republic (FFF – PLA filament)	(5.15 ± 0.55) × 10^3^ ÷ (4.87 ± 2.29) × 10^4^ (1 × cm^−3^)	CPC model 3,775 (TSI), SMPS model 3,936 (TSI)	Stabile et al. (27)
3DISON Multi 2; Rokit Inc., Seoul, Korea (FFF – PLA filament)	2.1 × 10^2^ ÷ 1.6 × 10^5^ (1 × cm^−3^)	SMPS model 3,910 (TSI), OPC model 3,330 (TSI).	Jeon et al. (26)
FlashForge Creator; FlashForge, Zhejiang, China (FFF – PLA filament)	∼4.5 × 10^4^ (1 × cm^−3^)	CPC model 3,776 (TSI)	Deng et al. (25)
model Form 2, Formlabs Inc., Somerville, MA and model NXE400, Nexa3D Inc., Ventura, CA (SLA)	1,73 × 10^3^ ÷ 2.10 × 10^3^ (1 × cm^−3^)	CPC model 3022A (TSI), FMPS model 3,091 (TSI)	Väisänen et al. (29)

CPC, condensation particle counter; FFF, fused filament fabrication; FMPS, fast mobility particle sizer; m-AMS, mini-aerosol mass spectrometer; PLA, polylactic acid; SLA, stereolithographic printer; SMPS, scanning mobility particle sizer.

Although there are no guidelines regarding individual exposure and as difficult as it is to compare our results with those of other studies (Table 3), one study (30) showed LDSA (μm^2^/cm^3^) values comparable to ours regarding for a printer using PLA.

The morphology and elemental composition of PM collected during presented here studies can be compared with previous studies. The presence of transition metal ions, e.g., iron was confirmed by the previous studies (28), and could be associated to metal ion presence in the printing polymer and printed filaments (31).

Limitations of this study relate to the lack of standardized industrial hygiene protocols with the use of 3D printers. There is no way to easily compare studies with each other because there is no uniformity in how data is collected. This particularly applies to the differing equipment used to monitor UFPs, since this is often expensive and difficult to obtain.

It would be useful to complement the data obtained with a detailed chemical analysis (both inorganic and organic constituents) of the collected NPs, since chemical composition is an important factor influencing their ability to cause harm. In addition, it would be valuable to investigate potential early health effect indicators related to nanoparticulate exposure.

## Conclusion

The use of 3D printing is growing rapidly both in industrial settings and everyday life. A few studies of aerosol dispersed particulate matter emissions during 3D printing activities are now available, and arouse concerns about hazardous exposure with health risks. Our pilot study showed that particulate matter of different size, morphology and elemental composition is emitted during printing activities. Although there are no reference limit values for UFP emissions, the average environmental nanoparticulate concentration in the laboratory using an SLA type printer exceeded the concentrations suggested by WHO guidelines.

## Data availability statement

The datasets presented in this article are not readily available because No dataset. Requests to access the datasets should be directed to lachowicz@unica.it.

## Ethics statement

Ethical review and approval was not required for the study on human participants in accordance with the local legislation and institutional requirements. Written informed consent from the participants was not required to participate in this study in accordance with the national legislation and the institutional requirements.

## Author contributions

GF: conceptualization, data curation, and writing–original draft. JL: investigation, methodology, and writing–original draft. SM: data curation and methodology. EC and LC: writing–review and editing. TC: data curation and investigation. MJ: supervision, validation, and writing–review and editing. MC: conceptualization, supervision, validation, and writing–review and editing. LL: supervision, validation, and writing–review and editing. All authors contributed to the article and approved the submitted version.

## Conflict of interest

The authors declare that the research was conducted in the absence of any commercial or financial relationships that could be construed as a potential conflict of interest.

The handling editor EO declared a past co-authorship with the authors JL, EC, LC, MC, and LL.

## Publisher’s note

All claims expressed in this article are solely those of the authors and do not necessarily represent those of their affiliated organizations, or those of the publisher, the editors and the reviewers. Any product that may be evaluated in this article, or claim that may be made by its manufacturer, is not guaranteed or endorsed by the publisher.

## Footnotes

^1^ https://www.dekati.com/products/elpi/

## Abbreviations

3D: Three dimension
AM: additive manufacturing
AQG: air quality guidelines
FFF: fused filament fabrication
NP: nanoparticle
PNC: particle number concentration
PLA: polylactic acid
SLA: stereolithographic printer
UFP: Ultrafine particles
VOCs: volatile organic compounds
WHO: World Health Organization
