# Supplementary material for: A pilot study of occupational exposure to ultrafine particles during 3D printing in research laboratories
Source: Front Public Health. 2023 Jun 2;11:1144475. doi: 10.3389/fpubh.2023.1144475 (PMC10272752; doi:10.3389/fpubh.2023.1144475)
Supplement: Supplementary file 3 [file Table_3.docx]

**Electronic supplementary material**

# **A pilot study of occupational exposure to ultrafine particles during 3D printing in research laboratories**

Giorgio Felici^1^, Joanna Izabela Lachowicz^1*^, Simone Milia^1^, Emanuele Cannizzaro^2^, Luigi Cirrincione^2^, Terenzio Congiu^1^, Mariusz Jaremko^3^, Marcello Campagna^1^, Luigi Isaia Lecca^1^

*^1^Department of Medical Sciences and Public Health, Division of Occupational Medicine, University of Cagliari, Cittadella Universitaria, 09042 Monserrato (CA), Italy*

*^2^Department of Sciences for Health Promotion and Mother and Child Care “Giuseppe D’Alessandro”, University of Palermo, 90127 Palermo, Italy*

*^3^Smart-Health Initiative (SHI) and Red Sea Research Center (RSRC), Division of Biological and Environmental Sciences and Engineering (BESE), King Abdullah University of Science and Technology (KAUST), Thuwal 23955-6900, Saudi Arabia*

1. **Technical data of 3D printer used in the Laboratory of the National Institute of Nuclear Physics.**

[Ultimaker S5 Pro Bundle Tecnical data.pdf](file:///C:\Users\acer\Desktop\Joanna\Running%20projects\NPs%20in%20Stamp%203D\Submission\Ultimaker%20S5%20Pro%20Bundle%20Tecnical%20data.pdf)

<https://manufat.com/download/Ultimaker-S5-PRO-TDS.pdf>

1. **Technical data of DiscMini**

[Scheda tecnica DISCmini](https://static-int.testo.com/media/ae/87/df0045b6f8dd/pb-testo-DiSCmini-Brochure-US.pdf)

<https://static-int.testo.com/media/ae/87/df0045b6f8dd/pb-testo-DiSCmini-Brochure-US.pdf>


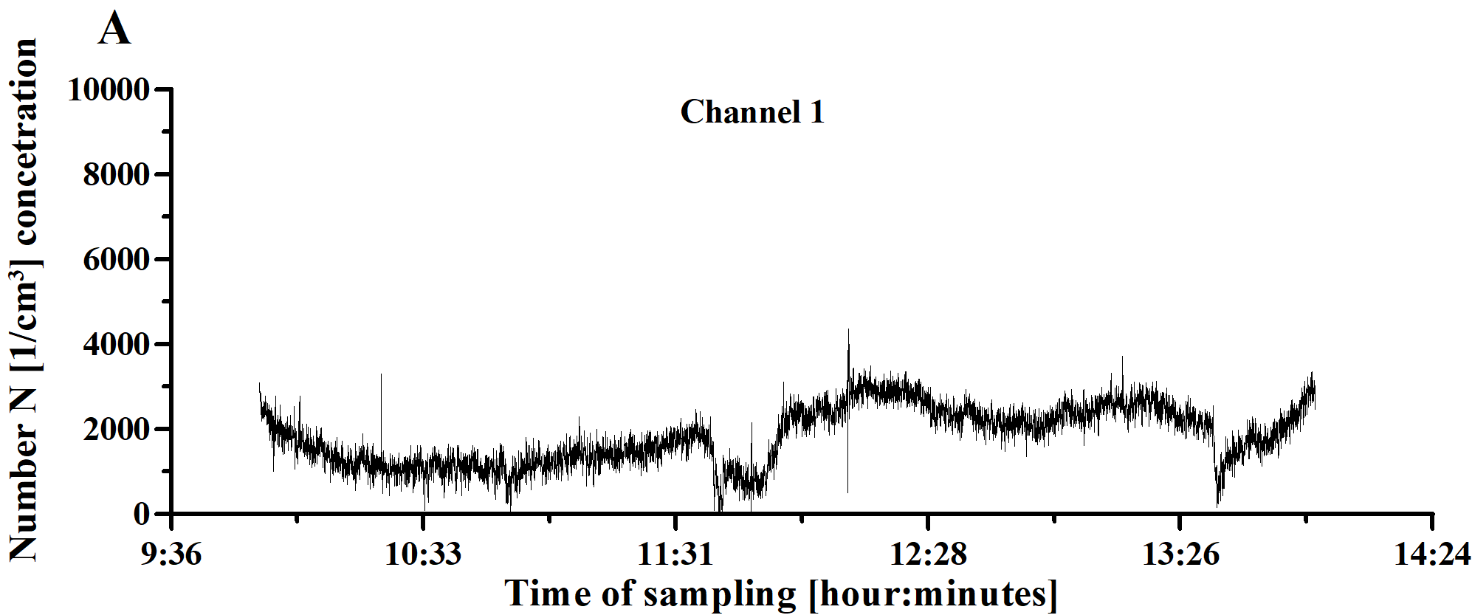


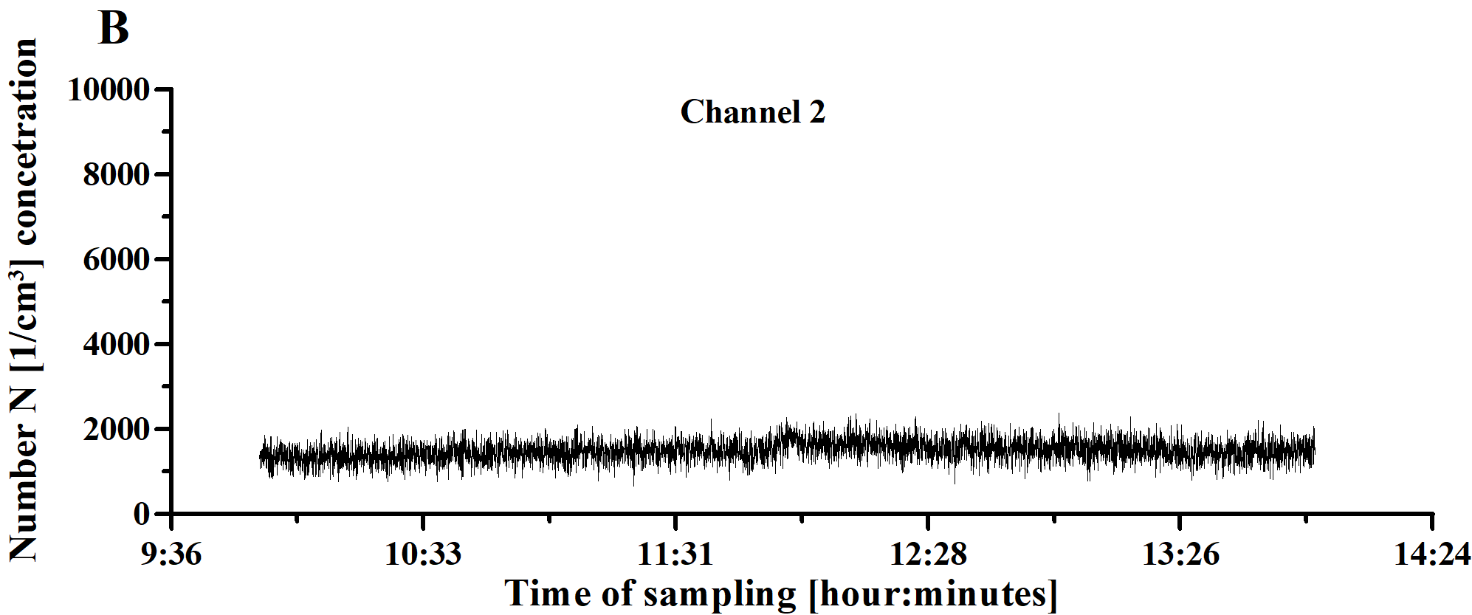


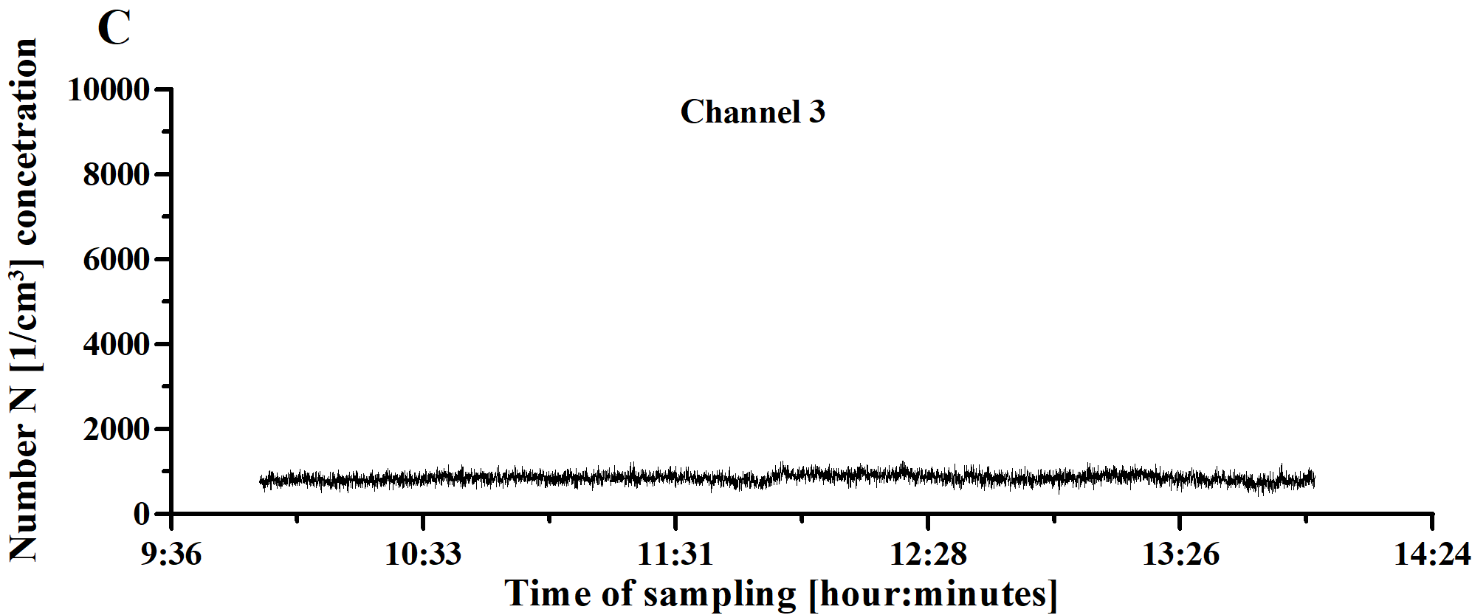


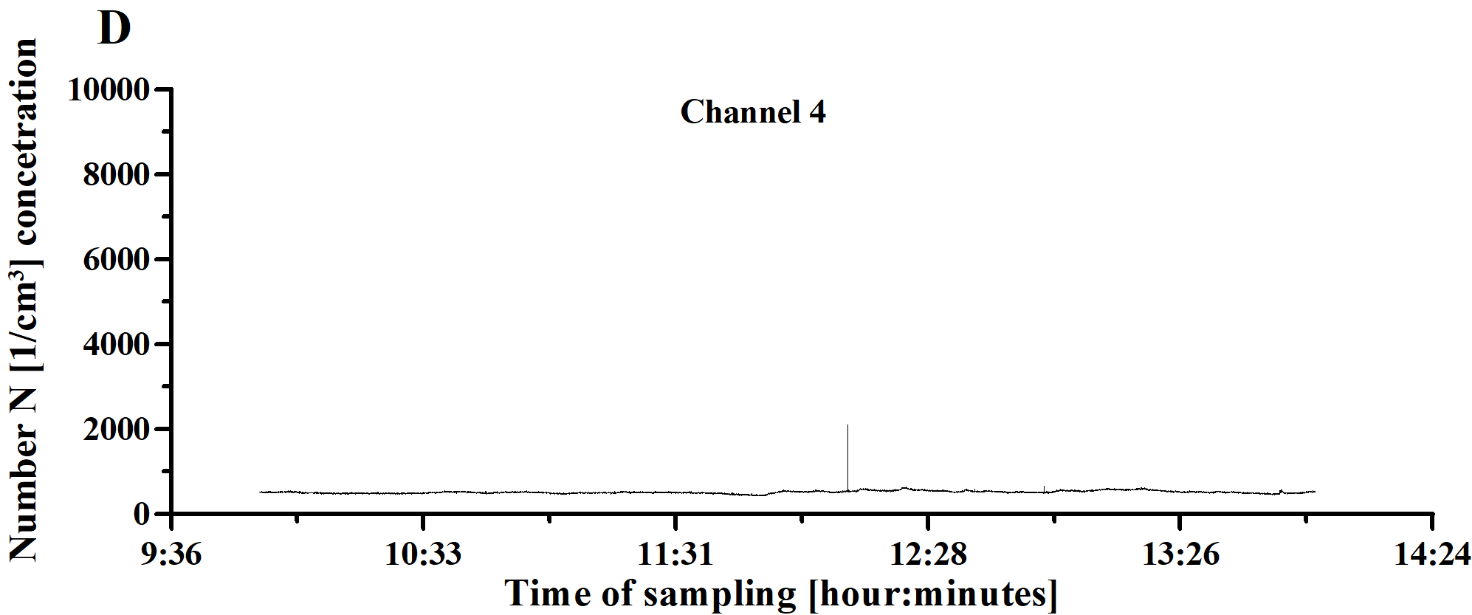


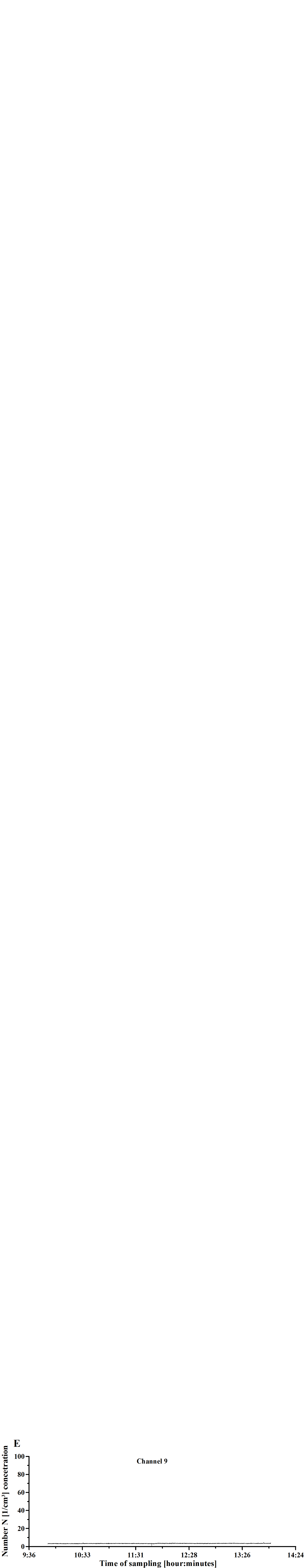


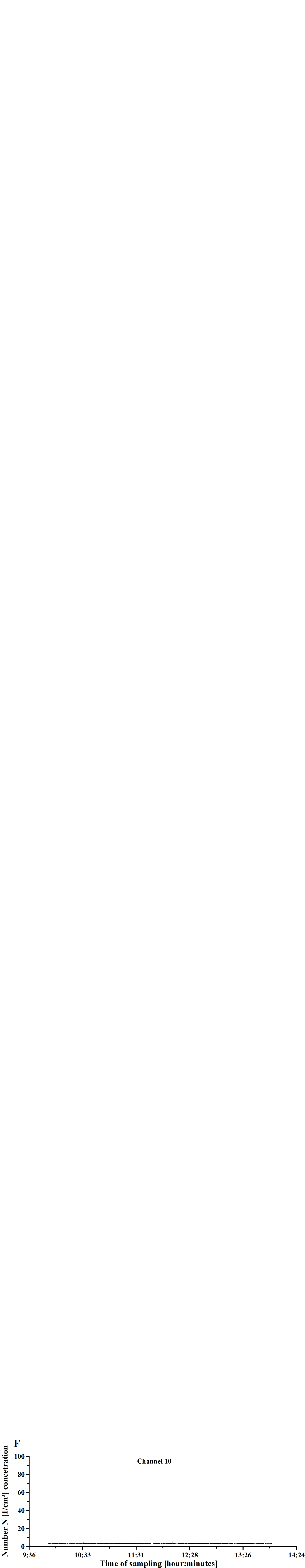


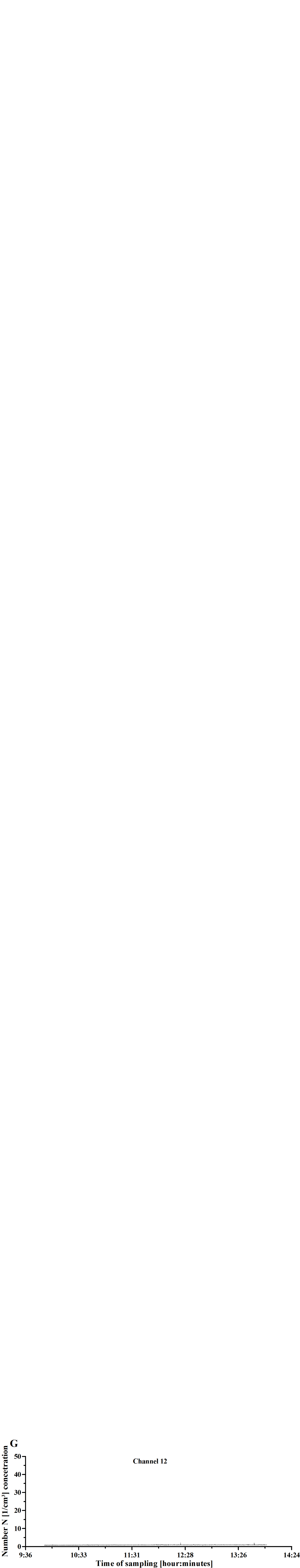


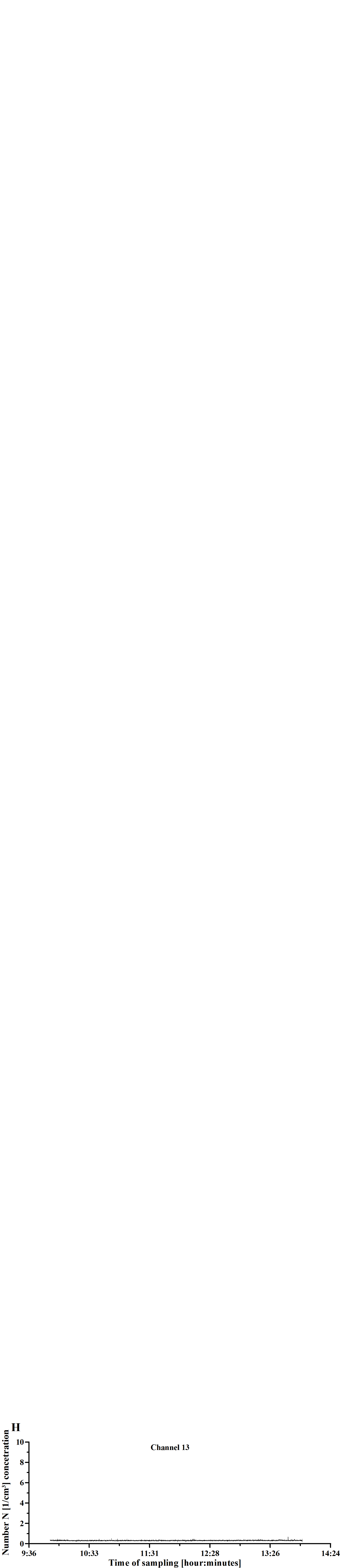


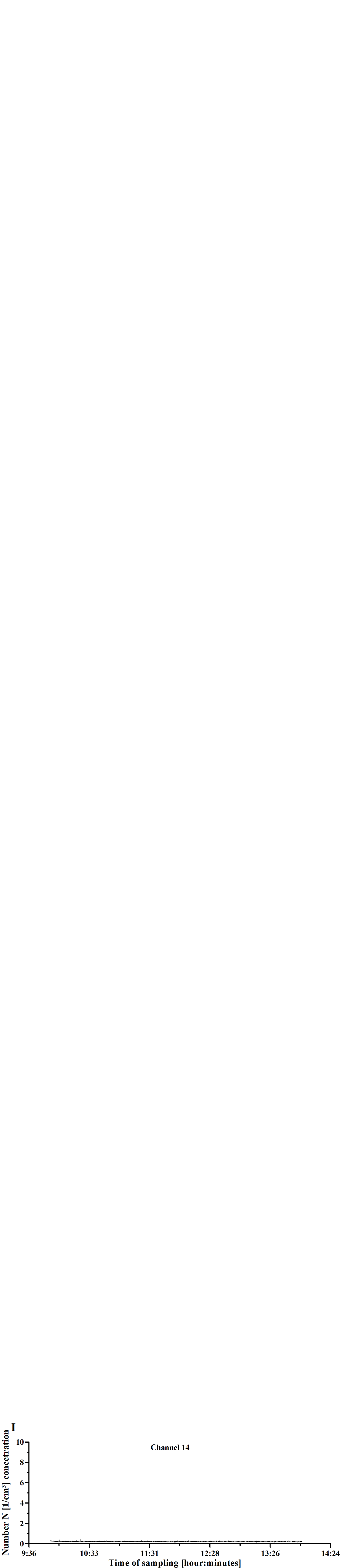


**Figure S1.** UFP concentration changes in the Einstein Telescope Laboratory setting, where 3D printing with fused filament fabrication was used. **A)** Concentration of particulate matter collected with channel 1. **B)** Concentration of particulate matter collected with channel 2. **C)** Concentration of particulate matter collected with channel 3. **D)** Concentration of particulate matter collected with channel 4. **E)** Concentration of particulate matter collected with channel 9. **F)** Concentration of particulate matter collected with channel 10. **G)** Concentration of particulate matter collected with channel 12. **H)** Concentration of particulate matter collected with channel 13. **I)** Concentration of particulate matter collected with channel 14.


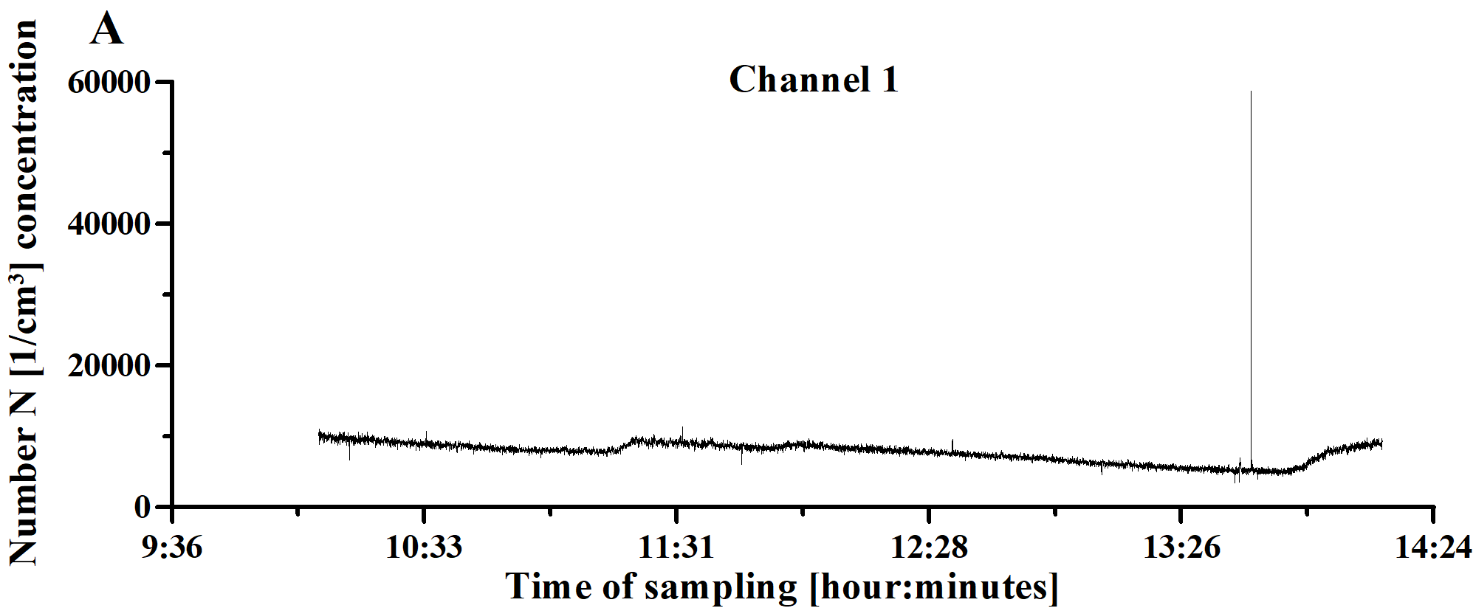


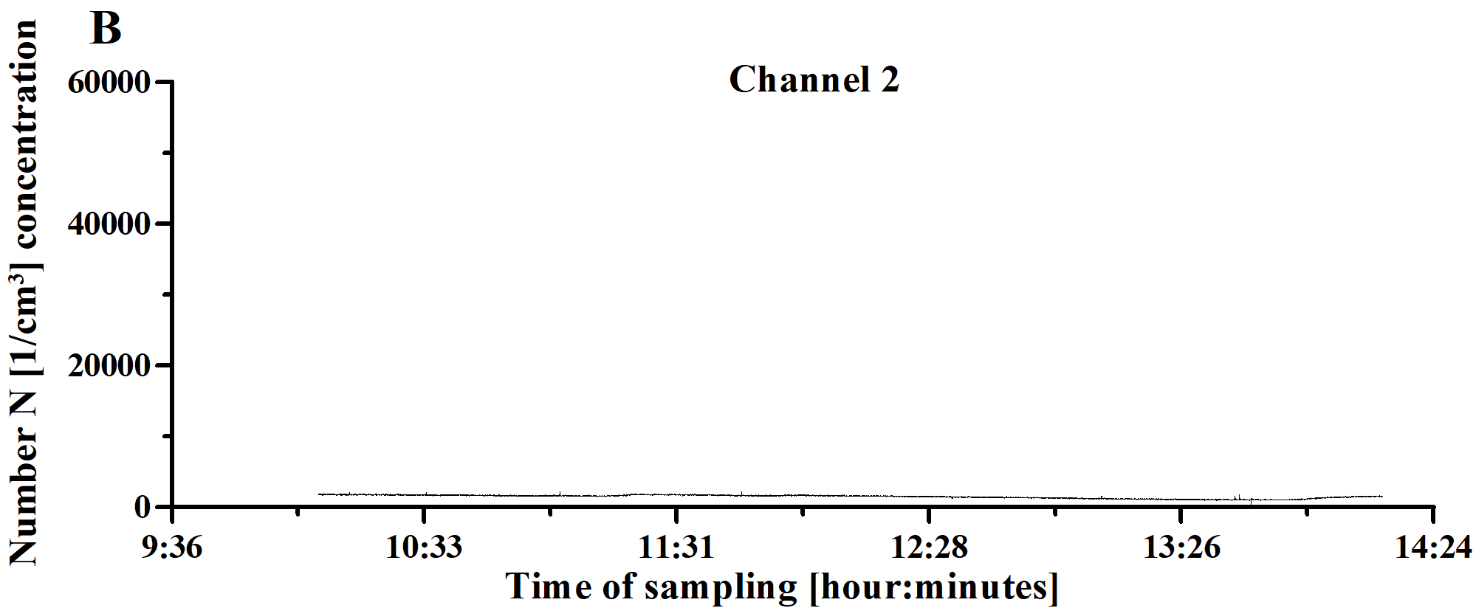


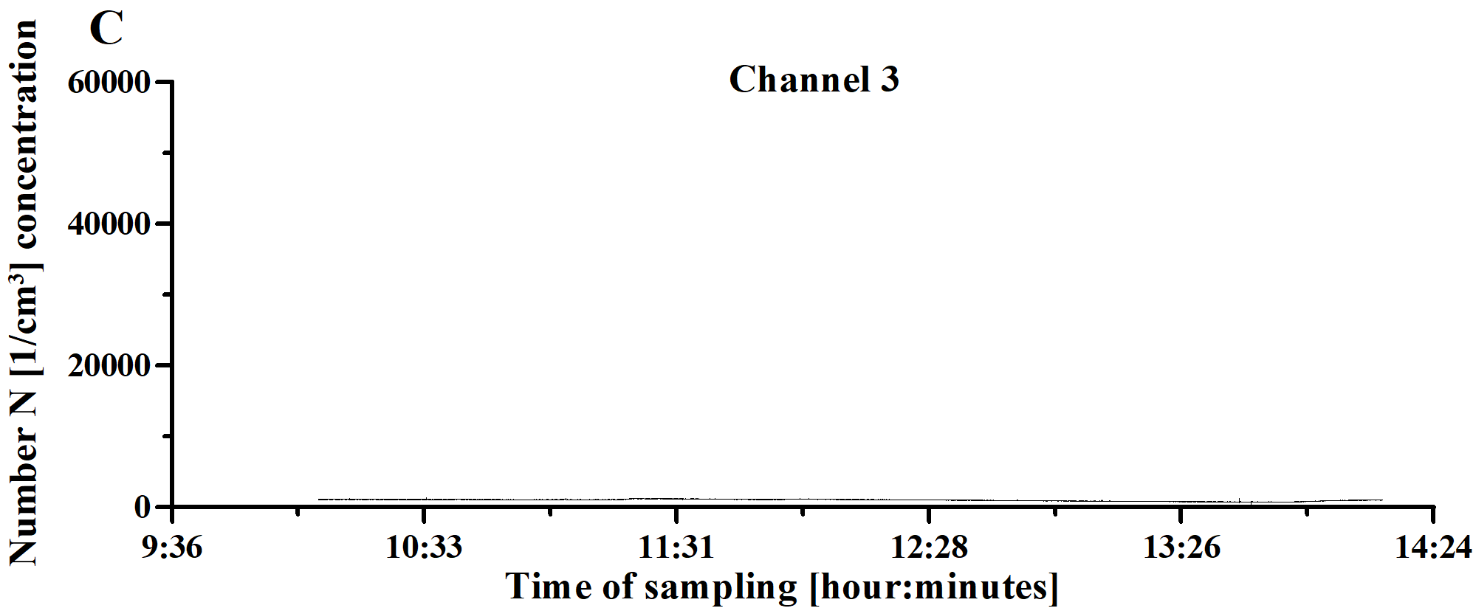


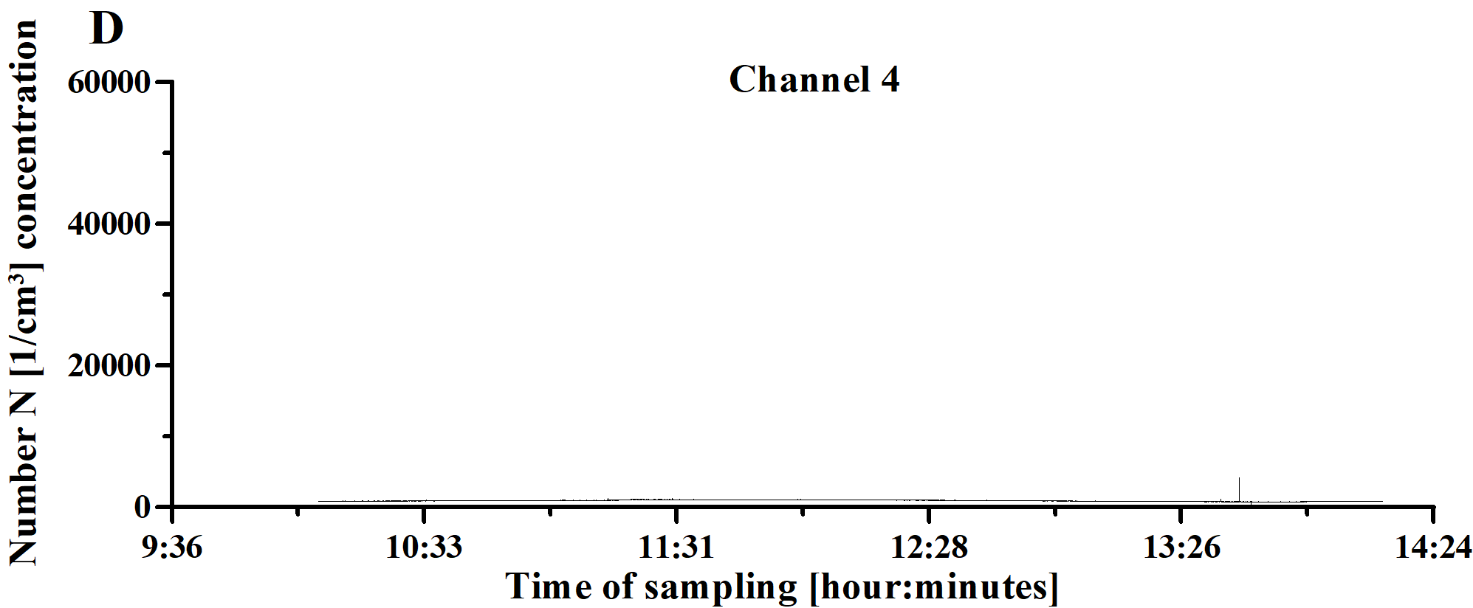


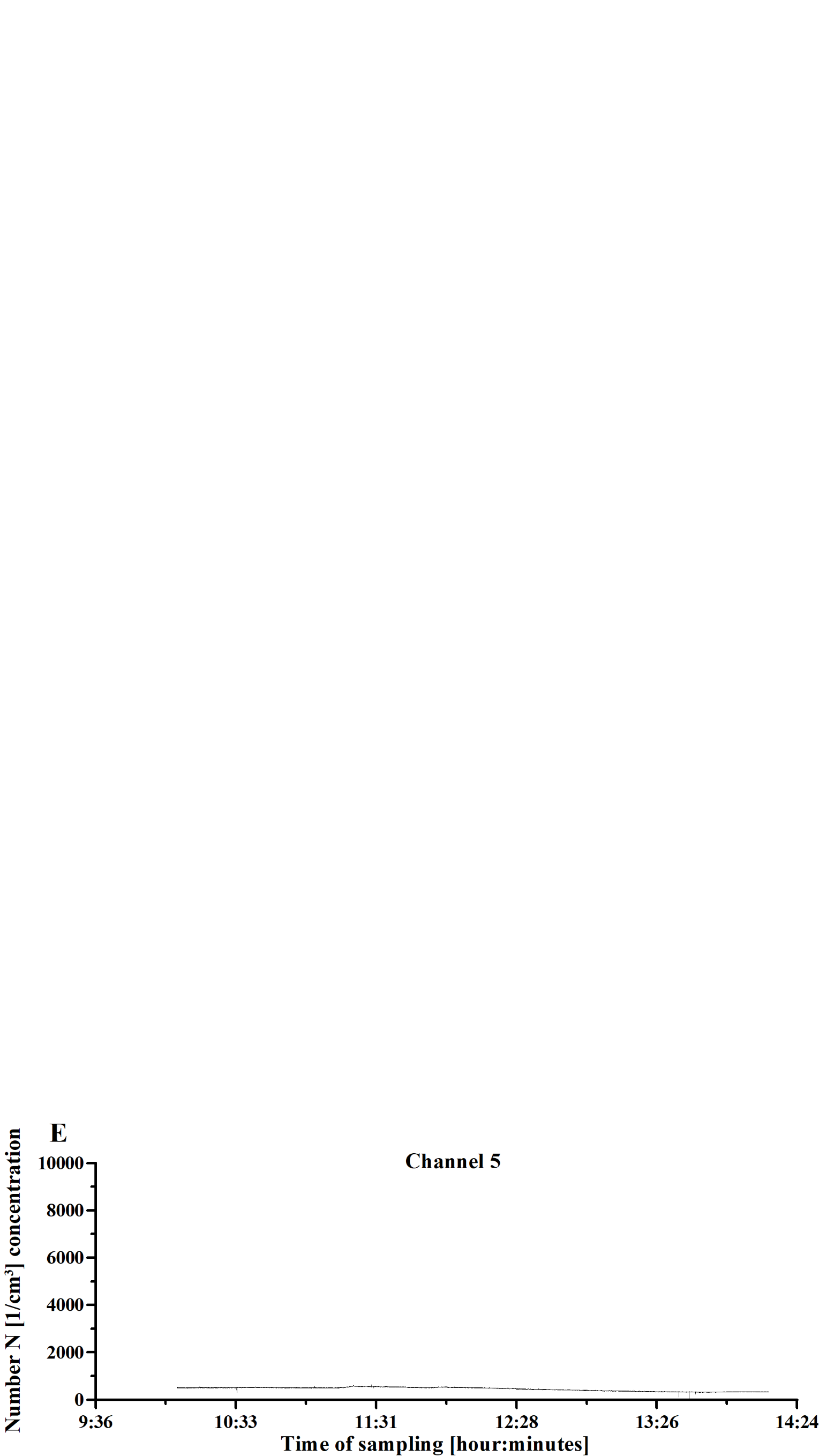


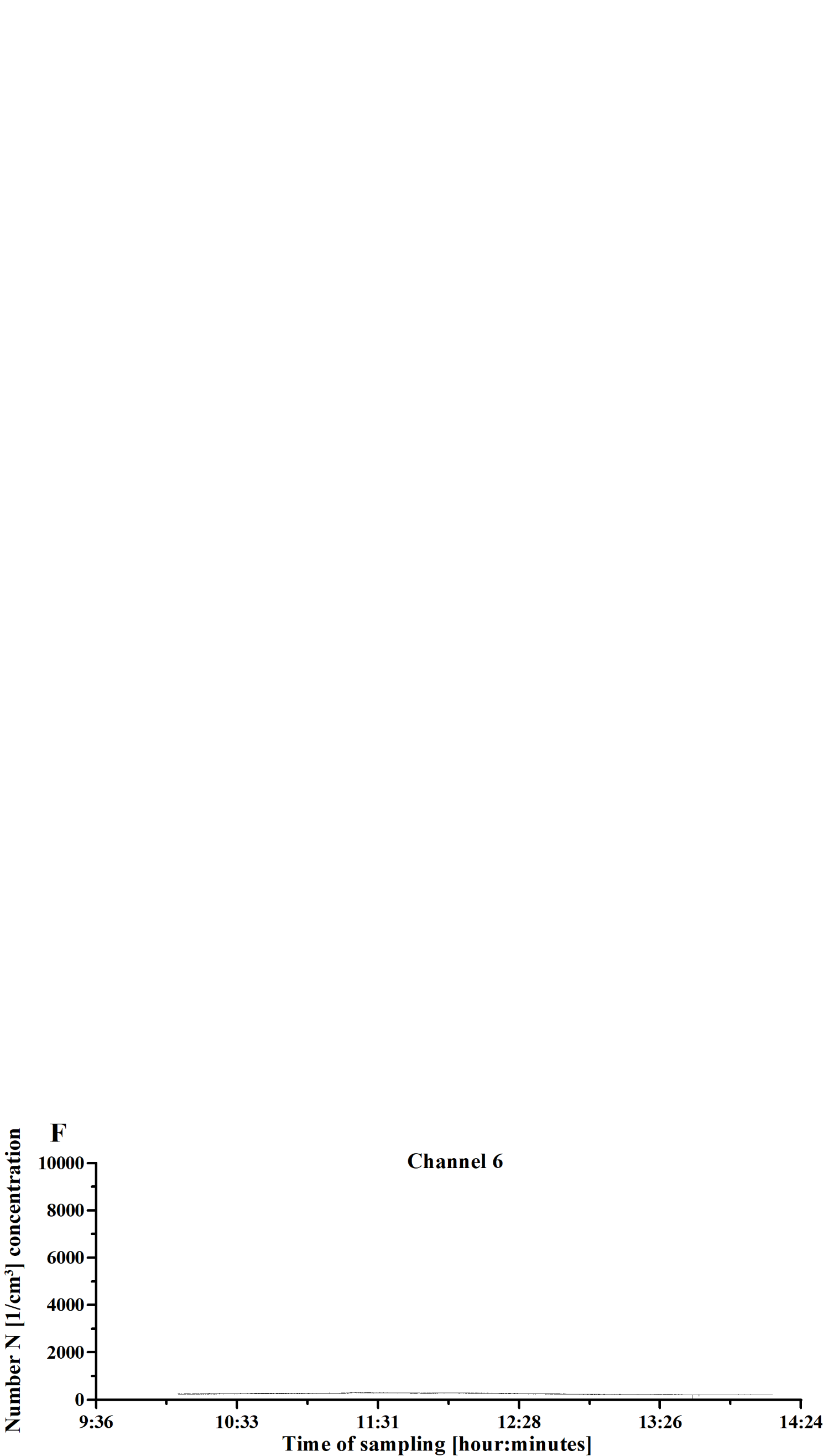


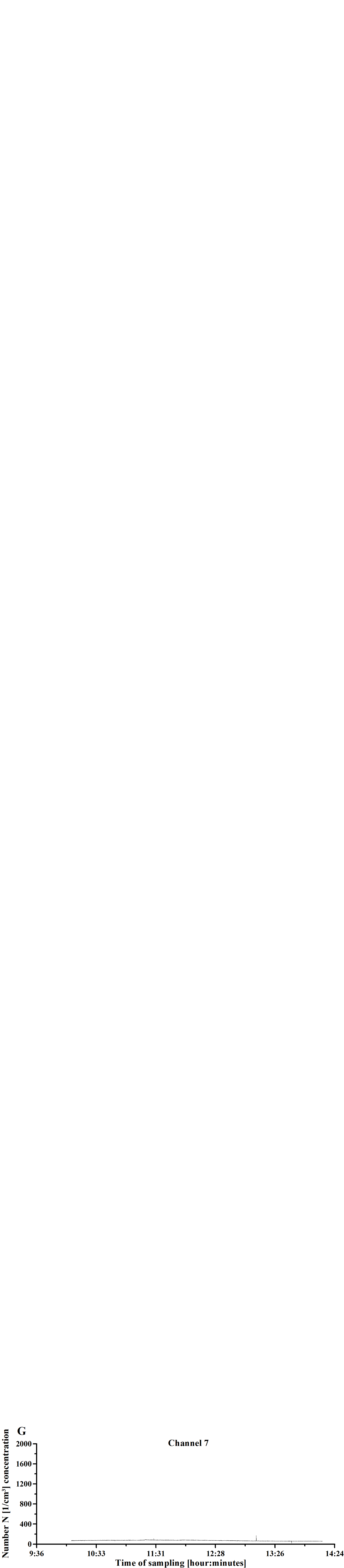


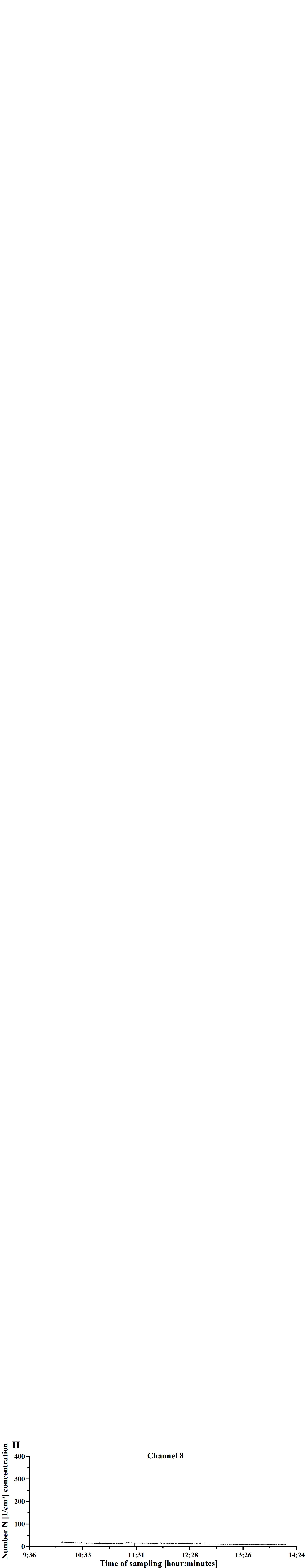


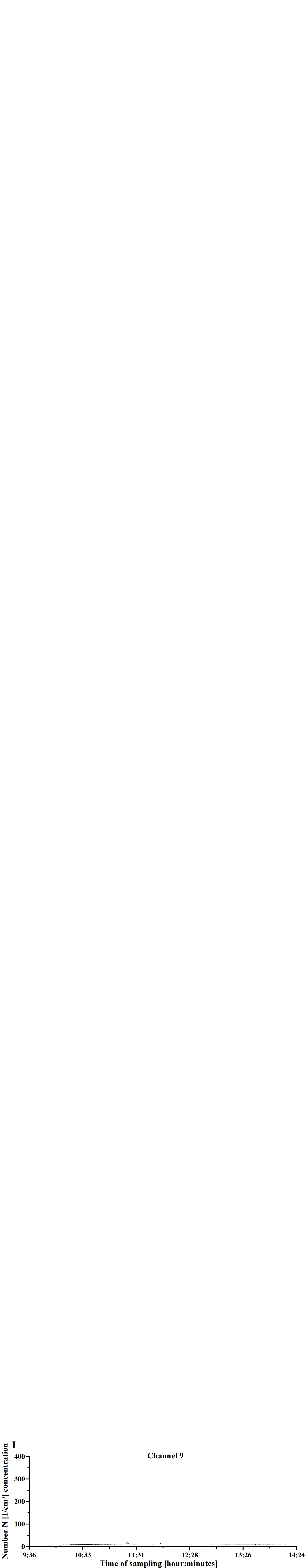


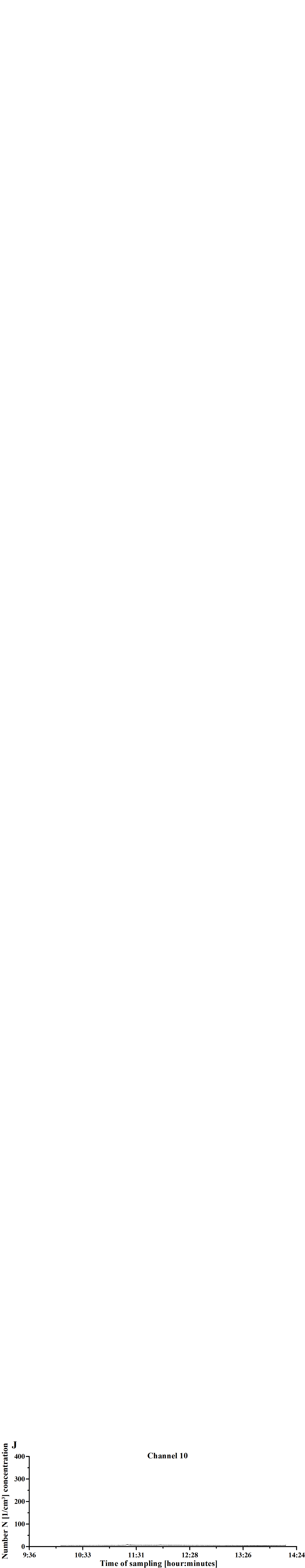


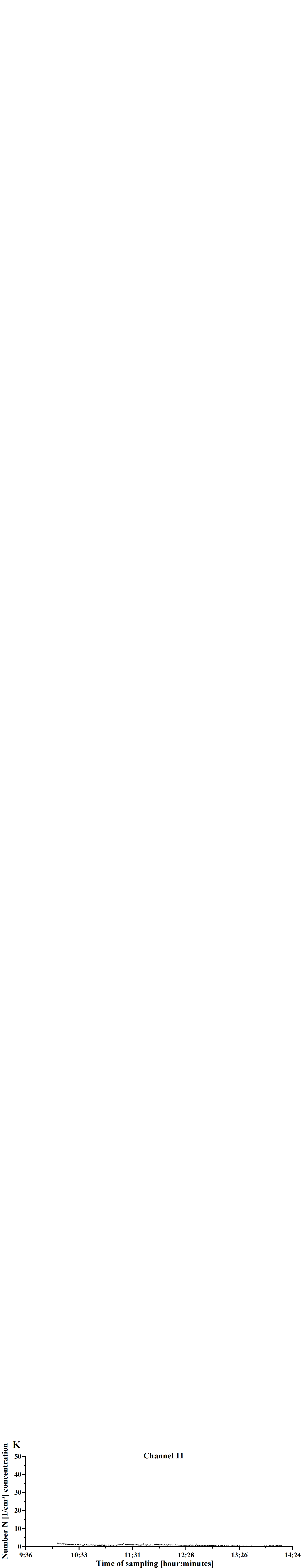


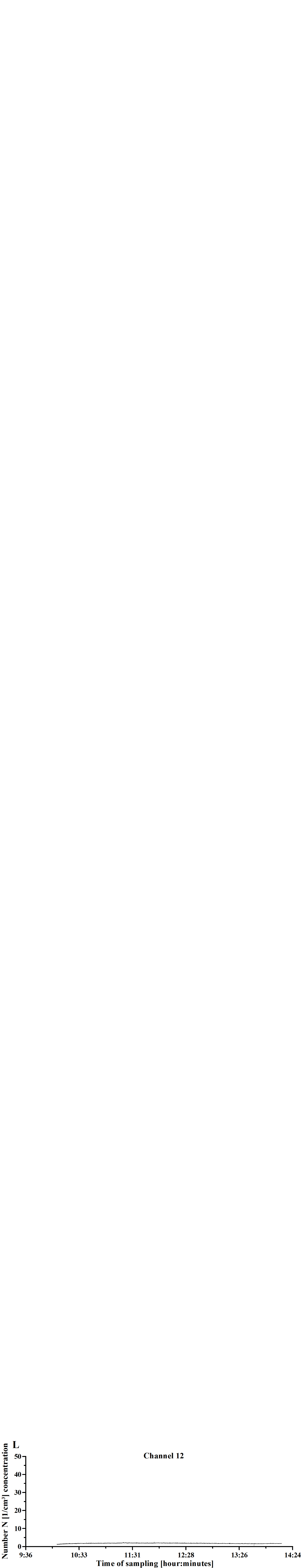


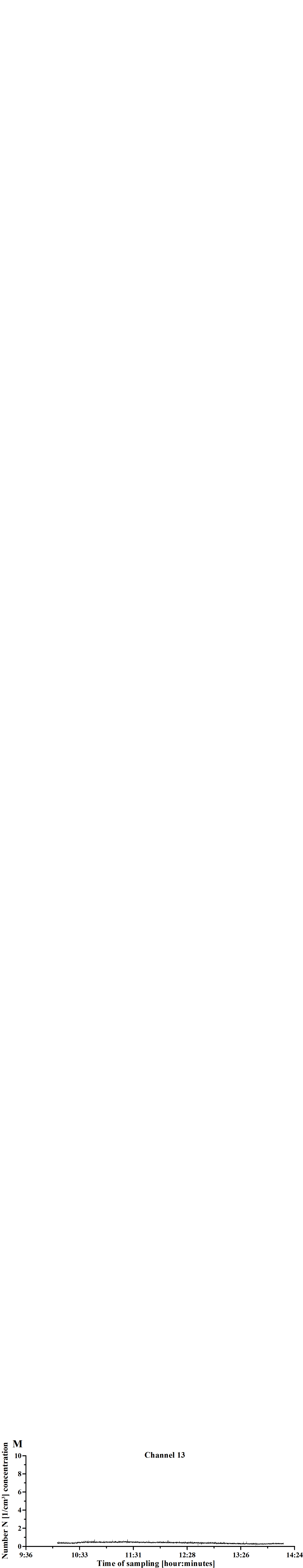


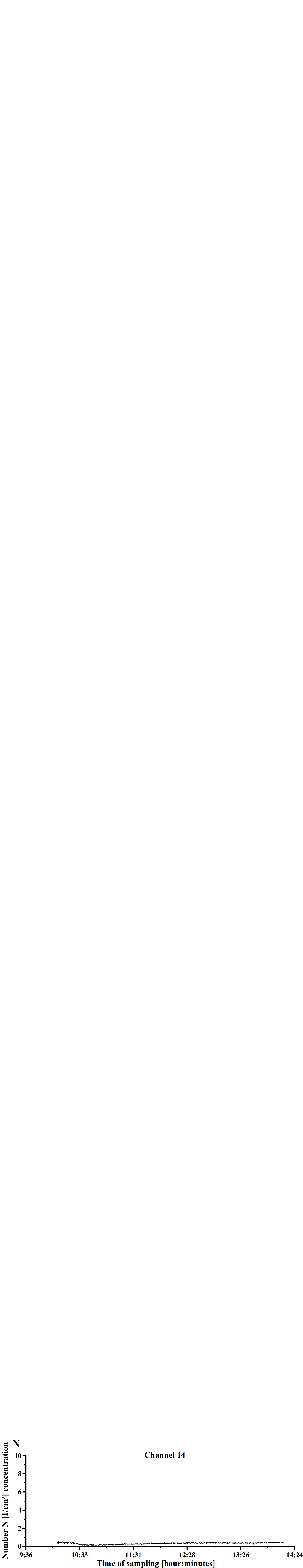


**Figure S2.** UFP concentration changes in the Department of Mechanical, Chemical and Materials Engineering at the University of Cagliari, where 3D printing was performed with polylactic acid biopolymers. **A)** Concentration of particulate matter collected with channel 1. **B)** Concentration of particulate matter collected with channel 2. **C)** Concentration of particulate matter collected with channel 3. **D)** Concentration of particulate matter collected with channel 4. **E)** Concentration of particulate matter collected with channel 5. **F)** Concentration of particulate matter collected with channel 6. **G)** Concentration of particulate matter collected with channel 7. **H)** Concentration of particulate matter collected with channel 8. **I)** Concentration of particulate matter collected with channel 9. **J)** Concentration of particulate matter collected with channel 10.

**K)** Concentration of particulate matter collected with channel 11. **L)** Concentration of particulate matter collected with channel 12. **M)** Concentration of particulate matter collected with channel 13. **N)** Concentration of particulate matter collected with channel 14.
